# Multiple memory systems, multiple time points: how science can inform treatment to control the expression of unwanted emotional memories

**DOI:** 10.1098/rstb.2017.0209

**Published:** 2018-01-29

**Authors:** Renée M. Visser, Alex Lau-Zhu, Richard N. Henson, Emily A. Holmes

**Affiliations:** 1 Medical Research Council Cognition and Brain Sciences Unit, University of Cambridge, 15 Chaucer Road, Cambridge CB2 7EF, UK; 2 Social, Genetic and Developmental Psychiatry Centre, Institute of Psychiatry, Psychology and Neuroscience, King's College London, London, UK; 3 Karolinska Institutet, Division of Psychology, Department of Clinical Neuroscience, Stockholm, Sweden

**Keywords:** intrusive memory, memory reconsolidation, translational science, aversive conditioning, post-traumatic stress disorder, psychological treatment

## Abstract

Memories that have strong emotions associated with them are particularly resilient to forgetting. This is not necessarily problematic, however some aspects of memory can be. In particular, the involuntary expression of those memories, e.g. intrusive memories after trauma, are core to certain psychological disorders. Since the beginning of this century, research using animal models shows that it is possible to change the underlying memory, for example by interfering with its consolidation or reconsolidation. While the idea of targeting maladaptive memories is promising for the treatment of stress and anxiety disorders, a direct application of the procedures used in non-human animals to humans in clinical settings is not straightforward. In translational research, more attention needs to be paid to specifying what aspect of memory (i) can be modified and (ii) should be modified. This requires a clear conceptualization of what aspect of memory is being targeted, and how different memory expressions may map onto clinical symptoms. Furthermore, memory processes are dynamic, so procedural details concerning timing are crucial when implementing a treatment and when assessing its effectiveness. To target emotional memory in its full complexity, including its malleability, science cannot rely on a single method, species or paradigm. Rather, a constructive dialogue is needed between multiple levels of research, all the way ‘from mice to mental health’.

This article is part of a discussion meeting issue ‘Of mice and mental health: facilitating dialogue between basic and clinical neuroscientists'.

## 1. Introduction

Your memory is a monster; you forget - it doesn't. It simply files things away. It keeps things for you, or hides things from you - and summons them to your recall with a will of its own. You think you have a memory; but it has you!

From: ‘A prayer for Owen Meany: a novel’ [1].

After psychological trauma, people can report vividly seeing the event unfold again in their mind's eye. For example, a person who was mugged at gunpoint while working at the cash till in a shop might see in their mind's eye vividly the gun pointed at them, the sunlight glance off the metal, hearing the sound of the safety lock clicking. This sensori-perceptual retrieval of an episode brings back with it the intense emotional experience of fear and the thought their life is about to end (for further examples, see table 1). These types of episodic memories are known clinically as ‘intrusive memory’—a core clinical feature of post-traumatic stress disorder (PTSD) and described in the *Diagnostic and Statistical* *Manual of Mental Disorders*, 5th edition (DSM-5) as ‘recurrent, involuntary, and intrusive distressing memories of the traumatic event(s)’ ([3, PTSD criterion B1; p. 271]). A critical aspect of this form of memory is that it springs to mind unbidden—that is, against the person's will. Thus, in the experimental memory literature, intrusive memories are a type of episodic memory that has been retrieved ‘involuntarily’ [4].

**Table 1.** Examples of intrusive memories from patients diagnosed with post-traumatic stress disorder. Examples taken from Holmes *et al.* [2].

‘The body between the tube and the platform’
‘Being pushed to floor, them saying “get down” and being tied up’
‘Gun put to head’
‘He runs off and I look back to my house to see my daughter crying and banging at door’
‘His face above me, laughing, laughing, laughing’

Involuntary retrieval is not restricted to episodic memories. Anxiety and stress-related disorders also involve automatic physiological responses, triggered either spontaneously or in response to (internal or external) threat-associated cues. For example, a person may experience increases in heart rate, muscle tone and perspiration when revisiting the location of an accident or assault, or when reminiscing about the event. Although one might not necessarily think of anxiety and stress-related disorders as ‘memory disorders’, this paper builds on the assumption that many of their symptoms are involuntary expressions of memory of previously experienced or imagined emotional events. Understanding what aspects of memory are maintaining the disorder may provide a way to progress much-needed treatment development.

To date, there is a good evidence base for the effectiveness of psychological treatments for both anxiety (e.g. obsessive compulsive disorder and social anxiety) and stress-related disorders (e.g. PTSD). Specifically, the types of psychological treatment recommended are forms of cognitive behavioural therapy [5–7] and, for PTSD, also eye movement desensitization and reprocessing (EMDR) therapy (e.g. [8]). While the UK national clinical guidelines [9–11] among others emphasize the aforementioned treatments, it is also noted that psychological treatment trial methodology has been a topic of debate [12]. Recently, some advances have been made with pharmacological treatments, specifically for anxiety disorders [13], although less so for PTSD [14]. It will be of interest to examine similarities and differences in mechanisms underlying effective treatments, regardless of modality (psychological or pharmacological) [12]. Overall, however, it is clear that even our best treatments need improvement: a substantial proportion of patients do not benefit from them, or experience a relapse after initially successful treatment (e.g. [15–17]). Furthermore, effective interventions to *prevent* and treat traumatic stress symptoms within the first hours and days after a traumatic event are lacking [18,19]. Bringing novel insights from basic science to the field of mental health, to understand mechanisms of change at a fundamental level, seems imperative to drive clinical treatment innovation [20]. Such insights may, furthermore, point at ways to make promising interventions more effective and widely applicable. Yet, translational science is challenging, in part because there is a ‘culture gap’ between basic and clinical science, with little interdisciplinary communication and collaboration, different language to describe the same phenomena, different journals to disseminate findings and thus limited knowledge of needs and discoveries in the other fields [20]. Aside from this gap, there are the methodological challenges associated with laboratory procedures, and boundary conditions, meaning that pioneering attempts to apply laboratory-developed interventions to clinical settings risk failure.

We believe that challenges to translational science arise, at least in part, from a lack of a shared vocabulary, which gives rise to a misunderstanding of clinically relevant targets at one end and insufficient appreciation of basic memory principles at the other end. We argue that, to successfully bridge findings ‘from mice to mental health’, it is essential to clarify (i) what aspects of emotional memory are clinical targets and (ii) when to assess and target these aspects.

We discuss the following questions:

— Why study emotional memory to understand anxiety and stress-related disorders?
— How to define emotional memory and targets for clinical interventions?
— How can the science of memory inform treatment innovation?
— What are the challenges of translational science from mice to mental health?
— How can neuroimaging in humans contribute to translational science?
— How could we facilitate the dialogue between basic and clinical science?

## 2. Why study emotional memory to understand anxiety and stress-related disorders?

Emotional memories are particularly resilient to forgetting [21,22]. This is not problematic *per se*. Long-lasting memories for aversive experiences can help us deal with similar situations in the future. However, aspects of emotion-laden memories can also form the basis of psychological disorders [23–25], particularly when memory retrieval is *involuntary.* ‘Intrusive memories’—typically of emotional events—are a form of involuntary recall and have been theorized to play a critical role in the development and maintenance of various psychological disorders [24–29], such as PTSD, and also including depression [30,31], bipolar disorder [32,33], social anxiety [34], agoraphobia [35], spider phobia [36] and health anxiety [37]. Involuntary memories may differ across disorders: intrusive memories in PTSD replay trauma content from an index episode; intrusions in agoraphobia have content related to agoraphobic themes such as being trapped—whether real or imagined; intrusive memories in bipolar disorder can have a future quality as if pre-playing a mania-inducing event. Although highly specific to the given disorder, what is common is their intrusive and imagery-based natures. Clearly, not all involuntary memories are pathological [38]—involuntarily recall can be useful at times—but when the intrusion contains highly aversive content, is unwanted and disrupts a person's functioning in daily life, then it is maladaptive and can enter the clinical domain. For example, Syrian refugees indicated that intrusive memories of traumatic experiences were linked to concentration problems and functional impairment on tasks associated with adaptation to the host country, such as language learning [39].

Here, we focus on memory in PTSD and, to a lesser degree, anxiety disorders. In PTSD, patients typically experience intrusive episodic memories of several distinct moments of the wider traumatic event [2]. That is, intrusive memories are not a replay of the whole event from beginning to end, but rather of a few fragments within it—known clinically as ‘hotspots’ [40]. Aside from these sensory episodic (predominantly visual) intrusive memories ([3, PTSD criterion B1; p. 271]), people with PTSD frequently exhibit increased heart rate, sweating and muscle tone in response to trauma-related cues, as well as impaired extinction of such responses. In the DSM-5, the diagnostic criterion B5 for ‘intrusion symptoms’ involves ‘marked physiological reactions to internal or external cues that symbolize or resemble an aspect of the traumatic event(s)’ ([3, PTSD criterion B5; p. 271]), while criterion E for hyperarousal symptoms involves ‘marked alterations in arousal and reactivity associated with the traumatic events(s) including hypervigilance (E3), exaggerated startle responses (E4) and problems with concentration (E5)’ ([3, PTSD criterion E; p. 272]). Both intrusive episodic memories and heightened physiological reactivity have been found to be predictive (among other factors) of the development of PTSD [41–43]. However, while general alterations in physiological reactivity are common across many disorders (e.g. most anxiety disorders), it is trauma-related intrusion symptoms (particularly criterion B1) that are the core clinical feature of PTSD (and acute stress disorder) and distinguish them from other disorders. Interestingly, evidence from experimental studies also suggests that, although physiological reactions in response to trauma reminders and intrusive episodic memories frequently co-occur, they do not seem to be associated with each other [44,45], which has implications for intervention development (see §3a).

As yet, it remains unclear whether it is better to focus on the ‘impact’ of a memory, or on the underlying memory itself. When an involuntary memory occurs, it can not only bring back the sensori-perceptual, emotional, psychophysiological and peri-traumatic cognitions described earlier (e.g. table 1), but it can trigger a cascade of other, more distal symptoms, such as further distress, non-specific hyperarousal symptoms, negative mood and cognitions, and unwanted avoidance. It is these cognitive and emotional reports of the immediate and more distal symptoms that are the typical focus of cognitive therapy. However, even exposure therapy procedures thought to focus more proximally on the trauma memory involve approaches that are thought to leave the original trauma memory unaltered [46], creating a new inhibitory memory trace instead.

Here, we argue that focusing on treatments that target the underlying memory more directly may be beneficial: controlling the expression of involuntary emotional memories may prevent the occurrence of the other symptoms altogether. Since the beginning of the century, a vast growing body of literature suggests that it is indeed possible to change the underlying memory trace. As explained below, the theory of ‘reconsolidation’ states that memories are not necessarily permanent [47–49], but can be updated, reduced or enhanced, under the right circumstances. As we shall discuss, this has inspired a whole new line of research looking at how the plasticity of memories can be used to modify clinically dysfunctional memories. But what exactly do we mean by ‘emotional memory’?

## 3. How to define emotional memory and targets for clinical interventions?

Studying memory is complicated by the fact that we cannot directly observe a memory trace, but have to infer it from the different ways it is expressed. In memory research, a variety of behavioural measures provide different ‘read-outs’ of information that has been stored, for example neural responses, peripheral physiology, actions or action tendencies, (subjective) verbal reports or explicit tests of memory. These responses are thought to be indices of some sort of underlying neural ‘engram’, the memory trace, which supports these responses. While in many situations different read-outs converge, below we argue that a distinction between multiple memory systems is useful and paramount to making translational progress.

### (a) Multiple memory systems

It is possible to show evidence of remembering in the absence of awareness of that remembering. For example, the Swiss neurologist Claparède [50] concealed a sharp pin between his fingers while greeting one of his amnesic patients with a handshake. Even though the patient reacted with surprise and anger, she forgot the encounter within minutes. However, when the neurologist tried to reintroduce himself shortly thereafter, the patient resolutely refused to shake his hand. She explained her reaction by stating that she was afraid that, perhaps, a pin was hidden in his hand, but even after repeated questioning could not remember that she herself had been stuck with it. While her brain was apparently able to form this association between a neutral handshake and a painful consequence, her brain was not able to voluntarily retrieve the event. The episodic part of the memory had not been stored.

Clinical cases like these, substantiated by abundant scientific evidence (e.g. [51–54]), teach us that our mind is made up of a constellation of agents that are, in principle, separable and rely on semi-independent circuits. In cognitive psychology, a classic distinction is made between ‘explicit’ or ‘declarative’ memory (what we can report, including episodic memory for events and semantic memory for facts) and ‘implicit’ or ‘non-declarative’ memory (including priming, reflexes and procedural memory such as aversive conditioning, motor skills and habits) (for a review, see [55]). Declarative memory is mostly subserved by the medial temporal lobe; most forms of non-declarative memory are subserved by subcortical areas such as the amygdala (aversive conditioning) and the striatum (skills and habits) [55]. These memory systems can be independently targeted [56–61] or damaged [62–65].

**Figure 1.**
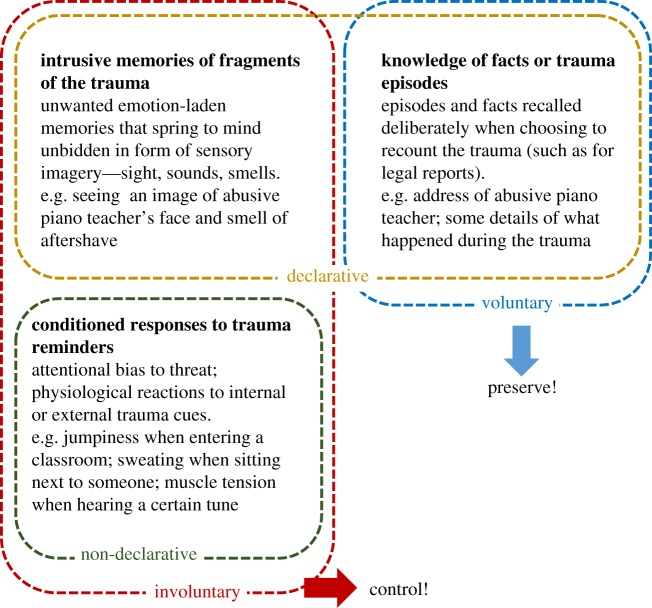
Diagram depicting how different memory systems may represent different aspects of the traumatic event(s), for example sexual abuse by a piano teacher. In general, clinically beneficial interventions should aim to target the maladaptive involuntary expression of trauma memories (e.g. intrusive memories), while preserving its voluntary recall (e.g. ability to testify in court). (Online version in colour.)

The idea that memory is composed of different systems is not new, but has now been widely accepted [66,67]. Furthermore, work in both non-human animals and humans has highlighted that certain types of memory can be modified, while preserving other types. Yet, when translating these findings to develop treatments for psychological disorders, relatively little attention has been paid to specifying what type of memory (i) can be modified and (ii) should be modified. We argue that rather than the traditional declarative versus non-declarative memory system distinction, the clinical focus should be on the intrusive nature of an aversive memory [68], which could, in principle, stem from both systems. A clinical intervention should thus seek to target the involuntary expressions of the memory (both declarative and non-declarative), without compromising voluntary attempts to access the same information or skill (figure 1). Voluntary forms of memory need to be preserved, e.g. for legal reasons a person may need to recount details of a trauma (eye witness testimonies and asylum reports). The simultaneous consideration of involuntary and voluntary retrieval of the same events should be a more central feature of traumatic stress research.

**Figure 2.**
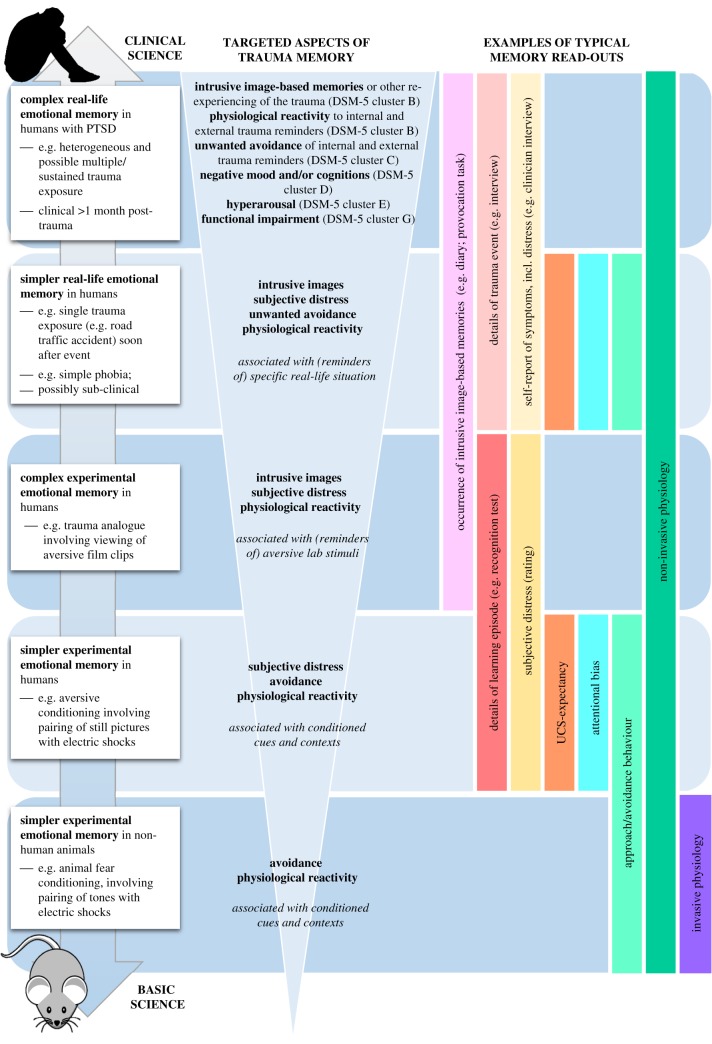
Examples of different aspects of trauma memory that can be targeted and associated research approaches along the translational research pathway(s) from mice to mental health. The left column shows the different levels at which trauma can be modelled. The middle column indicates which aspect of the (modelled) trauma may be relevant to target by an intervention, i.e. from a clinical perspective. The right vertical column highlights some of the memory read-outs that can be assessed at each level, with measures of declarative memory and subject reports being restricted to human research, and invasive physiology measures (such as structural at the synapse) being restricted to non-human animals. By and large most of the knowledge about timing and conditions for memory updating comes from the lower level, i.e. experimental research in non-human animals. Note that voluntary memory recall (e.g. details of the trauma) can be measured in humans, but is not the key clinical target of a treatment. DSM-5, *Diagnostic and Statistical Manual of Mental Disorders* [3]; UCS, unconditioned stimulus.

Successful translation of insights from basic science to inform clinical interventions (figure 2), depends on a clear conceptualization of what type of memory is being targeted, and how to model this. A widely used experimental model of traumatic stress responses and other types of aversive learning has been Pavlovian conditioning (box 1). Research using this paradigm in rodents shows that it is possible to selectively update one (aspect of) memory, without affecting another [72–74], and one response system (e.g. reducing freezing to a conditioned stimulus), but not another (alleviating suppressed reward seeking after conditioning) [75]. Similarly, many interventions based on this paradigm in humans show effects on one type of memory read-out (e.g. reduced startle reflexes in response to conditioned pictures), but not on other types (e.g. expectancy ratings of the likelihood of an aversive consequence) (e.g. [58,76,77]). Thus, it appears possible that rather than the whole memory, involuntary non-declarative aspects can be selectively targeted. Such dissociations are crucial when designing interventions for disorders such as PTSD: which aspects of memory can we modify, and is the aspect that can be modified, in fact, clinically meaningful?

Box 1. **Aversive conditioning paradigm: modelling involuntary and voluntary memory of threat associations in non-human animals and humans.**

The classic model to study simple associative learning and memory is Pavlovian conditioning [69], which is well suited for research across species [70,71]. In this paradigm, an initially neutral stimulus (conditioned stimulus, CS+; e.g. a triangle) is repeatedly paired with an intrinsically aversive stimulus (unconditioned stimulus, UCS; e.g. an electric shock), while another conditioned stimulus (CS−; e.g. a circle) is never paired with the UCS. With sufficient CS+/UCS pairings, the CS+ acquires the same aversive qualities as the UCS and will elicit a conditioned defensive response (CR) on its own. After repeated presentations of the CS+ without the UCS, the defensive response usually diminishes, a process that is referred to as ‘extinction learning’. In non-human animals, defensive responses are typically measured by assessing the amount of freezing in response to the CS, or avoidance behaviour; in humans, common measures include skin conductance responses, acoustic startle responses, heart rate, pupil dilation responses, action tendencies, UCS expectancies and subjective distress (figure 2).

Although aversive conditioning very precisely models the formation of associations between environmental stimuli and allows research across species, it does not model one of the core symptoms of PTSD: intrusive memories of the traumatic event. Involuntary retrieval of an intrusive memory is not the same as a conditioned physiological response: it (also) involves consciously retrieving a memory, i.e. drawing from the declarative memory system. Relatedly, physiological responses to trauma reminders are listed as a separate symptom from intrusive memories in the DSM-5 [3]; see earlier. Although a defensive response may very well be part of an intrusive memory, it does not account for its image-based episodic nature. Surprisingly, prominent neural circuitry models of PTSD based on aversive conditioning findings do not include mental imagery (e.g. [78]), which is a defining feature of episodic recall of an event. This conditioning literature, therefore, does not directly speak to the dissociation between involuntary and voluntary declarative memories.

An alternative experimental model with ecological validity for traumatic stress is the trauma film paradigm, which can model intrusive memories in response to viewing experimental material with traumatic content under controlled settings (for a review, see [79]; box 2). Emerging experimental interventions based on this paradigm have shown effects on one type of memory read-out (e.g. frequency of intrusive memories), but not on other types (e.g. recognition memory test) (e.g. [57]). Thus, it also appears possible that, rather than the whole memory, involuntary declarative aspects (e.g. frequency of intrusions) can be selectively targeted.

Box 2. **The trauma film paradigm: modelling involuntary and voluntary memory of psychological trauma in humans.**

The trauma film paradigm [79,80] has emerged as a well-established methodology to study intrusive memories of trauma, a core symptom of post-traumatic stress and one that can be distressing in its own right. It uses film stimuli in the laboratory, which contain traumatic content that can bring about intrusive memories subsequently in daily life. These memories are typically recorded in a diary, allowing for a frequency count of intrusive memories. Aside from intrusive memories, physiological reactivity and neural activity as well as subjective distress can be measured during and immediately after film viewing. This is similar to what is measured in conditioning paradigms (box 1; figure 2). Additional measures of voluntary memory include free recall and recognition of details of the film. Therefore, this paradigm enables the study of memory for more ecologically valid stimuli, but still within a laboratory setting, conferring additional experimental control.

Although the trauma film paradigm can only be used in humans, there are putative parallels with other types of memory read-out at ‘lower’ translational levels (e.g. distress ratings or psychophysiological measures in the conditioning paradigms; figure 2), which tentatively allow for translation of findings from basic neuroscience to simple real-life trauma. Yet, this is an understudied area. Given that assessment of intrusive memory frequency in humans typically involves ambulatory recordings over prolonged periods of time, research that directly compares intrusive memory frequency with other read-outs is sparse. The few studies that have assessed both physiology and intrusions in a laboratory setting indicate, however, a dissociation: trauma film reminder cues relative to control cues elicited both intrusive memories and heightened physiological reactions (e.g. skin conductance level) in one study [44], but the two measures were not associated [44]. Another study found more intrusions to film reminder cues, without heightened physiological reactivity (skin conductance and heart rate). Moreover, individual differences in cue-elicited physiological responses were not predictive of subsequent intrusive memory frequency in everyday life [45]. To translate findings from one memory read-out to another one in humans, future research should systematically examine under which conditions different read-outs converge or diverge.

**Figure 3.**
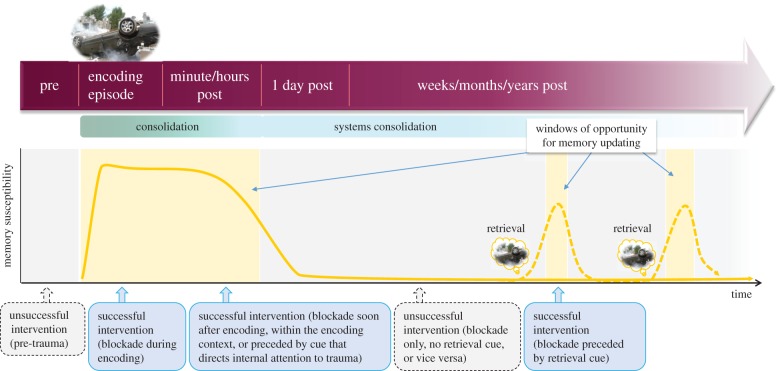
In the hours after an experience, memories are believed to go through an initial labile phase, before being stored into stable long-term memory, i.e. consolidation. The purple arrow depicts different time intervals with respect to the encoding of an aversive episode. Green gradients below indicate the putative processes of memory encoding and consolidation that occur during these different intervals, with systems consolidation referring to process by which memories become less hippocampus-dependent and integrated into a wider semantic network. Recent insights from studies in non-human animals suggest that (certain aspects of) memories are not necessarily permanent. Instead, they may become transiently malleable upon their reactivation, rendering them susceptible to interference or updating before returning to a fixed state, a process referred to as ‘reconsolidation’. This offers a second window of opportunity to interfere with consolidated memory (shown by yellow background shaded areas). Successful interventions (blue arrows) need to be timed such that the blockade interferes with memory when it is in an active, susceptible state (indicated by the dotted yellow line)—either in the first hours after an experience, or at later time intervals after a retrieval procedure (e.g. reactivation through reminder cues). In the first hours after an experience, blockade procedures may also need to be preceded by cues that orient attentional resources to the event in order for procedures to successfully interfere with it, for example when the intervention is delivered in a context other than the one in which the trauma occurred. Unsuccessful interventions, timed when memories do not yet exist, or are in a fixed state (i.e. not recently retrieved), are indicated by grey arrows with dotted outlines.

Not only is memory for a traumatic event likely to rely on different independent systems, but its nature also appears to change over time. In that light, the timing of any intervention is crucial. That is, it is important to appreciate that memory processes are dynamic, and that procedural details concerning timing are crucial when implementing a treatment, and again later when assessing its effectiveness (figure 3).

### (b) Multiple time points

The mind is not a video camera, automatically storing everything that occurs in front of the lens in a way that is easy to retrieve. To come back to the amnesic patient described by Claparède, she may be perfectly capable of remembering her neurologist's name and recognize his face in the short term, providing this information is actively rehearsed. Her failure to explicitly remember the incident with the pin does not indicate that she did not successfully encode the information. Rather, the information appeared not to be successfully consolidated in long-term storage, or lost in storage and therefore not successfully retrieved. Many people may respond with terror during a highly aversive experience, and will experience intrusions for a few days after, but only a few of them develop a disorder like PTSD where intrusions appear to continue for months or years.

A first time point to consider for memory modification is the ‘consolidation’ period: memory does not reach its final form during, or immediately after, encoding (unlike a video recording), but is still malleable for some time after the event. If altered during this malleable phase, the effects become apparent not initially, but at a later time points, after the memory has had a chance to consolidate [81]. The concept of memory consolidation has been most convincingly illustrated by experiments in which pharmacological manipulations administered immediately after encoding left short-term (4 h) retrieval intact, but induced full amnesia in the long term (24 h) [81,82]. Post-encoding processes account for this dissociation, as they induce the synaptic changes, including long-term potentiation (LTP), that are necessary for the stabilization of a memory trace in the hours after its acquisition [83,84]. This means that memory is initially malleable, i.e. there is the possibility to interfere with it.

While the duration of the consolidation window is typically estimated to be in the order of hours [85], another form of consolidation may continue indefinitely, with memories becoming more and more integrated into cortical networks [86,87]. However, this so-called systems consolidation happens mostly offline, when memory is in a stable state and not susceptible to interference (figure 3).

A second time point to consider for altering memories therapeutically is inspired by the theory of memory ‘reconsolidation’: findings from basic neuroscience indicate that consolidated memories can—upon their retrieval—enter a transient labile state, i.e. become malleable again [47–49] (figure 3). During this window, a memory can be updated, that is, new information can be integrated with a reactivated memory to modify future retrievals. For example, extinction learning within this window may diminish conditioned defensive responses more persistently than normal extinction learning, as the safety information has become part of the original fear memory [88]. The re-stabilization of a labile memory relies on a postulated process called ‘reconsolidation’. This process is dependent on *de novo* protein synthesis, and may be disrupted through pharmacological interventions (e.g. noradrenergic blockade [89]) or behavioural procedures that take away resources necessary for storage of certain elements of the memory (e.g. a competing visuospatial task [57]). To our knowledge, the precise time window of memory (re)consolidation has not been systematically investigated in non-human animals nor in humans, though is assumed to be in the order of hours [86]. The duration may depend on memory system and protocol used, and systematic work (e.g. using optogenetic manipulations to reactivate memory networks, followed by biochemical assays of plasticity markers) is needed to determine when exactly the window opens and closes. Again, the effects become apparent not initially (i.e. post-reactivation short-term memory is intact), but at a later time points after the (updated) memory has had a chance to reconsolidate [48,90,91].

The success of interventions that aim to harness principles of memory plasticity is dependent on their ability to target a memory when it is in an active, labile state. We have discussed two main time points during which memories can be altered: one that draws on theories of memory consolidation and one that draws on theories of memory reconsolidation. Inherent in this is the importance of a third type of time point: the outcome of memory modification cannot be observed until the memory has (re)turned to a stable state. This is important to consider when assessing the effectiveness of an intervention. Time has even further critical consequences: for example and fourth, a time gap of a few minutes may be needed between a retrieval cue and a blockade (figure 3), to allow a memory to destabilize (e.g. [88]). Fifth, pharmacological interventions require a precise understanding of temporal profiles of drug actions to target the memory when it is labile [92,93]. Research is clearly needed to define optimal time points for intervention delivery, which may be key to the success of a treatment.

## 4. How can the science of memory inform treatment innovation?

### (a) Successful translations

The principles of consolidation and reconsolidation imply that memory is malleable, either directly after an experience or upon reactivation [47–49]. This has inspired exciting lines of research aimed at modifying emotion-laden memory in humans, with the hope to develop relatively simple-to-deliver strategies that have the potential to interfere with involuntary recall of memories once they are established, i.e. hours, days, months or even years after the trauma (figure 3).

The first studies that translated findings in non-human animals [48,88,89] to humans used classical aversive conditioning to induce a simple emotional memory [94,95]. These showed that conditioned physiological responses could be eliminated by presenting a single reminder cue (the CS+) and administering a pharmacological agent (the beta-adrenergic antagonist propranolol [95]) or a behavioural intervention (an extinction procedure [94]) within the putative reconsolidation window. These findings have been replicated a number of times, using either the noradrenergic blockade [56,58,76,77,96–98] or a behavioural extinction procedure [99–103], with effects persisting for over a year (e.g. [58]) (for a discussion of non-replications, see section 4b ‘Challenges to translation’). Notably, these procedures only eliminated the automatic reflexive fear response (involuntary recall) and subjective feelings of fear, while leaving declarative knowledge about the aversive associations intact (figure 1).

Another line of research, which has drawn on theories of consolidation or reconsolidation (depending on the time window), uses the trauma film paradigm (box 2) to mimic complex emotional memory in non-clinical volunteers (figure 2). This has shown that the number of intrusive memories can be reduced, for example, by directly interfering with the molecular processes underlying memory consolidation at the receptor level. Nitrous oxide, also known as ‘laughing gas’, blocks *N*-methyl-d-aspartate (NMDA) receptors which are important for LTP. Individuals who inhaled nitrous oxide for 30 min immediately after trauma film viewing, compared with medical air (control condition), showed a steeper decline in the number of intrusive memories over the course of one week [104]. This suggests that across species, memory stabilization may rely on similar molecular processes (e.g. [90]).

Interestingly, a variety of interventions (whether pharmacological or behavioural/psychological or both) could, in principle, interfere with memory stabilization. For example, the frequency of intrusive memories can also be reduced by a relatively simple behavioural approach—by performing visuospatial tasks (e.g. Tetris; complex finger tapping), during a time period when memories are assumed to be labile (figure 3), i.e. either during trauma film viewing [105], within minutes to hours after film viewing [106–109], or upon their reactivation 24 h after encoding [57]. As expected, the task was not effective when administered before film viewing [110], or when only the task was given without retrieving the memory first [57]. The rationale behind this procedure is that the task taxes visuospatial working memory resources which are thought necessary to (re)store intrusive memories with strong visual components [108], acting as a ‘cognitive blockade’. In line with this notion, non-visuospatial tasks appear to be less effective or have no effect ([107,109], but see [111]). Crucially, when using a visuospatial task, across several studies the cognitive blockade selectively reduced intrusion frequency (involuntary recall), while leaving verbal and visual recognition (voluntary memory) intact (figure 1) (for a review, see [79]). As mentioned before, that a task could selectively reduce the number of intrusive memories while sparing voluntary memory of a traumatic event is desirable from a clinical perspective: someone would still be able to recall the factual course of events that constitute the trauma (e.g. recognize the perpetrator and testify in court), without having to fear being overtaken by unwanted intrusions of the trauma in their everyday life. Yet, the mechanisms behind this are still not fully understood and require further investigation [112].

Recently, findings from conditioning studies in non-clinical volunteers have been translated to emotional memory for real-life events in sub-clinical populations (figure 2). Three studies suggested that phobic responses could be decreased via reconsolidation-update mechanisms ([59], snake or spider phobia; [113], spider phobia; [114]). The procedure involved brief exposure to the object of fear (long enough to reactivate the memory, but short enough to prevent extinction; see below, section 4b ‘Challenges to translation’), followed by either a pharmacological manipulation (i.e. the administration of 40 mg propranolol [59]) or a behavioural manipulation (i.e. extinction training [113,114]). The manipulation changed avoidance behaviour into approach behaviour [59,113,114], with effects lasting up until three-month and 1-year follow-up. Importantly, the placebo group and the propranolol group without memory reactivation (i.e. no brief exposure to the spider) did not improve [59]. Also, in this case, different memory read-outs of the fear memory diverged (figure 2): initially, no differences were observed in subjective fear reports, but after three months, the reactivation + propranolol group scored in the normal range [59]. This suggests that effects of memory modification may require time to transfer to more distal symptoms, possibly via mechanisms other than memory reconsolidation (e.g. sufficient exposure to the previously avoided stimulus without the concurrent physiological and behavioural fear responses, leading to cognitive reappraisal of threat).

Findings from studies using the trauma film paradigm with non-clinical volunteers and cognitive task interference [106–109] have recently been translated to hospital settings, testing the modification of real-life emotional memory in the first few hours after a road traffic accident [115], and after traumatic child birth [116]—i.e. within the putative consolidation time window. Both early phase (proof of concept) clinical studies aimed to prevent the occurrence of intrusive memories of trauma, by having patients play the computer game Tetris soon after the event, as a way to interfere with the consolidation of the image-based components of the memory. In the road traffic accident study [115], a brief reminder cue was given prior to the cognitive task to orient the patient's memory to the accident, because patients were now in a hospital setting, whereas the accident had occurred in another context. In the traumatic child birth study [116], a reminder cue was deemed not to be necessary—mothers were in the same hospital setting context in which the trauma occurred as for the delivery of the intervention—and often with the baby. Both studies showed, as predicted based on studies with experimental trauma which used the same outcome measure [106–109], that the intervention group recorded significantly fewer intrusions in a diary that they kept in the week following the traumatic event compared with control groups.

Targeting simple phobias and recent real-life trauma memory (i.e. a prevention approach because this is before a clinical diagnosis of PTSD can be made) may be regarded as an early step of intermediate clinical complexity, compared with laboratory research in healthy individuals on the one hand, and the more severe anxiety disorders and PTSD on the other (figure 2). The more complex the memory becomes (e.g. older, or multiple events), the more important it is to define what aspect of the memory is being targeted and what read-out is used to assess whether it is effective (figure 2), and to ensure the timing of the intervention uses or induces periods of memory malleability (figure 3). Real trauma memories are typically stronger and broader than aversive memories formed in the laboratory. Case studies [117] and pilot studies [118], which combined a memory reactivation procedure and propranolol administration to block reconsolidation of trauma memories in patients with PTSD, seem to be promising, though many more are needed to understand potential limitations and non-replications (see next section). Such small-scale work helps to get the right ingredients to develop a new treatment. To test effectiveness of a treatment once it is there, various types of randomized controlled trials (RCTs) are necessary, but as argued below, at present more mechanistic insights may be required before investing in ‘expensive and time-consuming RCTs' [117].

### (b) Challenges to translation

While discoveries of memory plasticity fuelled excitement about the potential to offer brief interventions for mental health disorders with long-lasting effects, there has been considerable scepticism as well, especially among clinicians [6].

A first criticism that has been put forward is that the effects of the reconsolidation-update manipulation are usually restricted to one response system (for a review, see [6]). However, we argue to the contrary that this can be an asset therapeutically. While it is true that this means that an intervention does not wipe out an entire memory or miraculously ‘cures’ someone of a full disorder from one day to another, from a clinical point of view this does not mean it is not worth investing in. The elimination of one debilitating symptom may improve quality of life and functioning, and open up resources to tackle (or prevent) other symptoms, as well as have distal effects after time (e.g. [59]), and it may be possible to target different aspects of the memory one at a time. Moreover, as argued above, for trauma it is an advantage that manipulations are usually restricted to one response system—permanent erasure of all aspects of memory for a traumatic event is not the aim of evidence-based clinical interventions (figure 1) and could, in fact, bring about profound legal, ethical and clinical consequences of concern [119].

More broadly, specifying clear, precise clinical targets and memory read-outs allows one to systematically test and optimize interventions along the translational pathway (figure 2), which is impractical if one tries to treat an entire psychiatric syndrome at once. It is well recognized that the current classification system for mental health disorders, DSM-5 [3], poses challenges for research owing to ongoing debate about the precise symptoms that constitute a given disorder and the overlapping symptoms across different disorders [120–122]. Thus, a ‘precision focus’, focusing on one symptom rather than a whole disorder, may be more helpful when considering the aetiology of disorders. Indeed, arguably, it is the confusion that arises when talking about syndromes rather than symptoms that can stagnate scientific progress across various areas of mental health.

Second, scepticism has been fuelled by the fact that initial attempts to apply procedures in the clinic have been difficult to replicate. For example, the initial finding that trauma cue evoked physiological responding could be reduced following a pharmacological procedure that was postulated to tap into reconsolidation mechanisms [118,123] was not replicated in a follow-up study [124]. It should be noted that both studies had several important limitations, such as small sample sizes [118,124] and lack of control groups for either the effects of reactivation [118] or general drug effects [124]. It is important to follow this up and delineate boundary conditions that arise during the complexity of clinical translation. Failed replications are not unique to clinical settings: pharmacological [125] and extinction procedures within the reconsolidation window [97,126–129] have sometimes not replicated—though note that there are more successful replications than non-replications. Furthermore, there is evidence that the procedure does not work strongly enough to eliminate all involuntary recall for everyone [115,130]. Occasional (or partial) non-replications are to be expected at this early stage and yield important clues to direct future lines of research.

An important question is whether studies that failed to find an effect managed to actually trigger reconsolidation. Experimental studies have highlighted so-called boundary conditions—circumstances under which memory updating cannot take place (for a review, see [6]). The first one is cue and context specificity: the reminder situation that is used to reactivate the memory may need to be identical to the encoding situation [73], which may be difficult to achieve in clinical practice. However, other experimental studies have found effects of memory modification extending from one reactivated element to other elements of a compound memory [74], to similar—but not identical—stimuli, including imagined threat events [76,77], and to persist across contexts [77,88]. Furthermore, the finding that interventions in sub-clinical populations were successful in contexts other than the original encoding contexts [59,115] suggests that this is not such an issue, as long as the targeted memory system is sufficiently engaged. Another boundary condition is the finding that reactivation of a memory is necessary, but not sufficient, to destabilize a memory. Whether or not a memory trace is destabilized may, at least partly, depend on whether there is something to be learned, i.e. whether novel or conflicting information requires an update [56,96,98,131,132], highlighting a potential evolutionary function of reconsolidation mechanisms [86,133]. This may be governed by prediction error, i.e. a mismatch between what is expected and what occurs (for a computational account of when and how prediction may play a role in memory updating, see [134]). For example, one study [56] showed that in the case that a certain stimulus (CS) always predicted a shock (UCS), a single unreinforced presentation of the CS led to a violation of predictions and triggered memory destabilization. However, in the case of uncertainty (e.g. the stimulus was only reinforced in 50% of the trials during encoding), more unreinforced trials were needed to create a mismatch with what was expected [56]. Similarly, older and stronger memories may require additional retrieval procedures for updating to take place (e.g. [135,136]), as do individual differences such as exposure to chronic stress and high trait anxiety [130,137].

Thus, the optimal timings (see the earlier section (3b) ‘Multiple time points') and duration of the reactivation procedure are likely to depend on the precise encoding history, with more ambiguous memories possibly requiring more conflicting information, longer durations or possibly multiple retrieval instances. Yet, longer and repeated retrievals of a memory bear the risk of initiating extinction learning (which involves the creation of a new, inhibitory memory trace [46]) instead of memory destabilization. When this happens, no updating appears to take place [98,138–140]. Some clinical pilot studies [118,123,124] have used script-driven imagery for the reactivation of the trauma memory, similar to EMDR-like treatments: the question is whether the method of retrieval used in these treatments (prolonged, and with no obvious violation of expectancy) would have triggered reconsolidation, or instead extinction learning. Furthermore, memory read-outs during an intervention should not be mistaken for clinical targets. For example, a clinician may be inclined to continue a reactivation procedure until a decline in physiological arousal or subjective distress in response to trauma cues is observed. Yet, such a focus on acute fear relief may be counterproductive, and by inducing extinction learning, in fact prevent modification of the original trauma memory [98,138–140]. This would have enormous implications for clinical practice, suggesting that therapists should not merely rely on seeing direct within-session change, but should prioritize capturing longer-term effects, i.e. after their therapy session has ended.

This touches on an important point: we do not currently have an index for memory destabilization or reconsolidation, other than inferring it retrospectively from the strength of memory recall. But this is essentially circular: if we observe a behavioural change, we infer that the memory was modified; if not, we conclude that some of the criteria for updating were not met. While, in an experimental setting, overt expectancies have been used to indicate at a group level whether memory destabilization was achieved [56], this index may not be sensitive enough to apply in real time on an individual level, as patients may not always be able to voice what they fear and what event violates their expectation. What we need is a memory read-out for offline processes that underlie memory plasticity.

## 5. How can neuroimaging in humans contribute to translational science?

Neuroimaging—especially (functional) magnetic resonance imaging (fMRI)—has been frequently used for studying memory processes because of the possibility to predict which elements of an encoding episode will later be remembered and which will later be forgotten. So-called subsequent memory paradigms [141] have yielded several markers that predicted the later voluntary recollection of information [142], or the involuntary retrieval of experimental trauma [143–145]. Recent conditioning studies showed that patterns of neural activation during encoding can also be used to predict the long-term expression of involuntary non-declarative memory (i.e. physiological responses to conditioned stimuli) [146,147]. Furthermore, neuroimaging has been used to study memory reactivation and offline consolidation processes directly, without having to interfere with them [148–152]. Perhaps, in time, techniques such as fMRI could help us capture critical time frames for memory updating—a process for which no other read-out exists. Such a read-out may not be feasible for use in clinical practice (yet), but it would allow us to test out procedures in real time that trigger memory destabilization, without having to retrospectively infer it from behaviour.

Another way that neuroimaging could advance translational science is by checking whether neural circuitries underlying a certain read-out of memory (e.g. startle responses) are comparable across species, or across non-clinical and clinical human populations. If so, there is stronger theoretical reason to translate interventions that target that read-out in non-human animals to humans, and eventually to clinical populations. If not, it may be necessary to go back a step and focus on a different read-out, until these ‘intermediate phenotypes' yield sufficient overlap to proceed. By covarying different memory read-outs within and across different translational levels (figure 2), we may be able—in a ‘Sudoku’ way—to bridge gaps across paradigms and species. Neuroimaging could provide a valuable tool to build these bridges.

## 6. How could we facilitate the dialogue between basic and clinical science?

Harnessing the science of memory plasticity seems promising for the innovation of treatments to control the expression of unwanted emotional memories. Yet, to capture emotional memory in its full complexity, including its malleability and its context-dependent activation, science cannot rely on a single method, species or paradigm. Indeed, work on memory updating, including reconsolidation, has been conducted in different species (e.g. crabs, mice, rats and humans), different paradigms (e.g. classical context and cue conditioning, trauma film paradigm, as discussed here, but also operant conditioning, motor tasks) and different modalities (vision, audition and olfaction). A constructive dialogue between different levels of research is difficult as long as clinicians do not familiarize themselves with basic science, and scientists do not go beyond formulating the implications of their findings as merely ‘offering opportunities for the treatment of disorder ‘X’’. Both sides need to communicate and bear the challenges that will ensue in order to make progress [20]. Here, we end with two recommendations.

1. Across translational steps, it is important to have a clear conceptualization of what aspect of memory is being targeted (or not) and how different memory systems map onto core clinical symptom(s).

Scientists need to be clear what memory system they are assessing and manipulating in their experiments, and how it links to clinical phenomena (figure 2). Clinicians, in turn, need to feedback which clinical phenomena are most relevant to target and are core to a given disorder. For precision, it can be an advantage to focus on one symptom instead of a whole disorder, because it is virtually impossible to model a complete syndrome in the laboratory, especially when there is less clinical consensus than desirable about how symptoms should be clustered in the first place.

2. It is important to appreciate that memory encoding and retrieval refer to dynamic constructs and that many aspects of timing are crucial, both when designing and implementing an intervention, and when assessing its effectiveness.

Memory is not permanent, but can be updated (figure 3). A range of factors determine whether updating will actually take place. Factors that need to be taken into consideration for treatment include the time since the traumatic event (e.g. immediately post-trauma or later), the time of diagnosis/symptom assessment relative to the intervention (memory read-out), the duration of the consolidation window as well as the reconsolidation window, and the similarities between encoding situations and retrieval cues. To systematically establish timing parameters for an intervention and avoid circular reasoning, an independent (real-time) read-out of memory susceptibility is urgently required. Neuroimaging may be a promising tool to provide such a read-out and validate translational steps across different levels (figure 2).

With the challenges raised here, one can imagine that it is demanding during the treatment of established emotional memories to define various key parameters of an intervention, including (i) what retrieval cue represents the key aspect of the trauma memory that needs to be changed, (ii) how to present this retrieval cue to destabilize a memory, (iii) how to select an intervention to interfere with a destabilized memory, (iv) how to find the optimal timings to deliver such an intervention, and (v) how to tailor treatment to an individual, i.e. taking into account that the stimulus or procedure that triggers memory plasticity in some individuals may not be optimal for others. It should be noted that some of these challenges (e.g. selecting appropriate retrieval/reminder cues) do not exclusively apply to (re)consolidation-based treatments, but also apply to existing psychological treatments that seek to reduce established trauma symptoms (e.g. exposure therapy and imagery rescripting). Rather than setting challenges aside as definite boundary conditions, we are actually encouraged by the rapid accumulation of knowledge that is advancing our insights about necessary and optimal conditions for effective memory modification. In turn, ‘back-translation’ studies of effective procedures, in practice, may yield invaluable cues for basic research. We think that further progress can be made by investing in frameworks that reduce obstacles in the translational path from mice to mental health.

An effective dialogue to facilitate translational research is dependent on regular meetings and funding opportunities that allow memory scientists to consult with clinicians and *vice versa*. Scientists often seem unaware that evidence-based psychological treatments target processes of emotional learning and memory. Similarly, experimental work in the laboratory seems abstract and remote to clinicians working with patients with complex trauma. A constructive dialogue is not just important for the field of PTSD and anxiety—maladaptive emotional memory is core to many other psychological disorders. A focus on transdiagnostic processes and single symptoms opens up new opportunities for precision compared with a focus on full disorders. For example, intrusive memories occur frequently in depression [30] and in substance-use disorders (albeit with different content), while cue-induced responses (e.g. craving, approach tendencies evoked by drug paraphernalia) can be reduced by similar reconsolidation-update manipulations as those used in phobias [153,154].

If memory is like a monster carrying traumatic information, it may be time to tame it. A first step is to familiarize ourselves with its many heads, each responding to its own triggers and each producing its own sights and sounds. We may come to realize that not all of the heads are ugly and not all of the sounds are terrifying. We can live with the monster, as long as it is not jumping out unexpectedly, carrying the negative emotions, physical stress and sensori-perceptual fragments that impair functioning.

## Data accessibility

This article has no additional data.
